# The mitochondrial and chloroplast genomes of *Lagerstroemia suprareticulata* revealed a convergent genome morphology in genetic material evolution

**DOI:** 10.3389/fpls.2026.1746941

**Published:** 2026-02-25

**Authors:** Bo Qin, Xin Huang, Rihong Jiang, Yaoheng Huang, Kaidao Sun, Jianling Li, Gangmin Zhang

**Affiliations:** 1 Laboratory of Systematic Evolution and Biogeography of Woody Plants, School of Ecology and Nature Conservation, Beijing Forestry University, Beijing, China; 2 Guangxi Key Laboratory of Special Non-wood Forest Cultivation & Utilization, Guangxi Laboratory of Forestry, Guangxi Forestry Research Institute, Nanning, China

**Keywords:** evolution, horizontal gene transfer, *Lagerstroemia*, mitochondrial genome, structure evolution

## Abstract

To investigate the mitochondrial genome characteristics and evolutionary dynamics of *Lagerstroemia suprareticulata*, we performed complete assembly and annotation of its mitochondrial genome, followed by comparative genomic analyses with related species. This research presents the initial comprehensive mitogenome of *L. suprareticulata*, a 364,645 bp independent single cyclic structure with a whole average GC content of 46.20%, twice the size of the chloroplast genome and an approximately similar tetrad structure. It comprised 62 functional genes and 386 open reading frames. Besides two long repeats above 800 bp, simple sequence repeat analysis revealed a predominance of mono-nucleotide and tetra-nucleotide repeats, which is consistent with patterns observed in most Lythraceae species. A total of 480 C-to-U RNA editing sites were predicted in 36 protein-coding genes, with the highest number in *nad4*. AUG and UGG had a relative synonymous codon usage value of 1, while GCU had the highest RSCU (1.62). *ccmB* and *rps4* may have undergone positive selection, whereas *atp8* and *cox1* experienced strong purifying selection. Phylogenetic analysis based on mitochondrial and chloroplast genomes confirmed a close relationship between *L. suprareticulata* and *L. indica*. Collinear segments decreased with increasing evolutionary distance, and gene rearrangement analysis revealed a lineage-specific gene arrangement pattern in *Lagerstroemia*. Homologous sequence analysis identified 34 mitochondrial-chloroplast homologous sequences (accounting for 4.63% of the mitochondrial genome) and 2182 mitochondrial-nuclear homologous sequences. These results provide a foundation for understanding the mitochondrial genome evolution of *Lagerstroemia* and Lythraceae, and may offer valuable genetic resources for horticultural and evolutionary studies.

## Introduction

*Lagerstroemia* constitutes a genus of angiosperms that has garnered substantial scholarly and practical attention within horticultural and ecological research paradigms (Fang et al., 2019; Zhou et al., 2023b; Qiao et al., 2025). Characterized by their vibrant, prolonged floral displays, extensive cultivar diversity, and adaptability to heterogeneous climatic regimens, these taxa have emerged as foundational elements in landscape architecture, urban afforestation initiatives, and ornamental horticultural practices globally (Yue et al., 2024, 2025). Beyond their aesthetic utility, *Lagerstroemia* species fulfill critical ecological functionalities, contributing to biodiversity maintenance, edaphic stabilization, and the provisioning of niches and trophic resources for pollinators and faunal communities (Adhikari et al., 2019). Furthermore, select species within the genus have been subjected to phytochemical and stress physiology investigations, underscoring their potential as reservoirs for pharmaceutical compounds and stress-resistant genetic determinants, thereby holding significance for agricultural biotechnology and pharmacognosy research (Yu et al., 2022). *L. suprareticulata*, a deciduous tree or shrub in this family. It is endemic to the southwestern region of Guangxi, China, listed as endangered level in the Threatened Species List of China’s Higher Plants. This species flourishes in limestone regions, demonstrating potential for use in the greening of rocky mountains and ecological rehabilitation. Additionally, it bears fragrant blossoms, an uncommon feature within the *Lagerstroemia* genus (Zhou et al., 2023a).

Mitochondria, often designated as the “cellular powerhouses,” are indispensable organelles primarily responsible for adenosine triphosphate (ATP) synthesis via oxidative phosphorylation (Meyer et al., 2019; Carillo and Ferrante, 2025; Wang et al., 2025). However, their biological significance transcends energetic metabolism, as mitochondrial genomes (mitogenomes) exhibit extraordinary structural complexity and evolutionary plasticity (Wu et al., 2022). These genomic features—encompassing large molecular size, frequent intragenomic rearrangements, expansive intergenic regions, and recurrent horizontal gene transfer events-distinguish them from the relatively conserved nuclear and plastid genomes (Zhou et al., 2023c). Such unique attributes render mitogenomes invaluable substrates for investigating evolutionary processes, including species identification, speciation mechanisms, adaptive divergence, and phylogenetic reconstruction (Chen et al., 2017; Najer et al., 2024). Recent advancements in high-throughput sequencing technologies have catalyzed a paradigm shift in organelle genomics, facilitating the rapid and cost-efficient *de novo* assembly and annotation of mitogenomes across a broad spectrum of plant lineages (Zou et al., 2025). This technological progression has precipitated a proliferation of comparative mitogenomic analyses, which have elucidated evolutionary patterns and mechanistic processes governing genome diversification (Wang et al., 2024). Nevertheless, despite the accumulating body of knowledge, mitogenomic characterization remains conspicuously limited for numerous plant genera, including *Lagerstroemia*. To date, only fragmented sequence data and incomplete assemblies have been reported for a handful of *Lagerstroemia* species, impeding comprehensive elucidation of the genus’ mitogenomic architecture, genetic variability, and evolutionary trajectory.

Given the confluence of ecological, horticultural, and scientific imperatives associated with *Lagerstroemia*, a comprehensive mitogenomic analysis represents a critical knowledge gap requiring redress. Such an investigation would not only augment the broader framework of plant mitogenomics but also yield mechanistic insights into evolutionary dynamics within the genus and its phylogenetic relationships with cognate taxa (Van de Paer et al., 2017). Specifically, *de novo* characterization of complete *Lagerstroemia* mitogenomes would enable the systematic identification of coding sequences, repetitive elements, and structural variants—fundamental components for deciphering mitochondrial functional biology, inheritance mechanisms, and evolutionary trajectories. Furthermore, comparative mitogenomic analyses between *Lagerstroemia* and related genera within the Lythraceae could reveal conserved and divergent patterns of genome evolution, including gene gain/loss events and rearrangement dynamics, thereby refining phylogenetic resolutions within the family (Wang et al., 2023). This, in turn, would contribute to the development of robust evolutionary frameworks for interpreting angiosperm diversification. Additionally, mitogenomic investigations may yield practical applications in horticultural science, such as the identification of molecular markers for marker-assisted selection programs targeting stress tolerance or ornamental trait enhancement. In the present study, we aim to generate, assemble, and annotate the complete mitogenome of *Lagerstroemia suprareticulata.* Through detailed analyses of genomic architecture, gene content, and sequence polymorphism, we seek to address the following research objectives: (1) What are the defining features of the *Lagerstroemia* mitogenome, including size, gene repertoire, and organizational structure? (2) What classes of repetitive sequences and structural variants are present, and how do they contribute to genomic complexity? (3) What phylogenetic insights can be derived from mitogenomic data regarding the placement of *Lagerstroemia* within the Lythraceae? By addressing these objectives, this research endeavors to advance our understanding of *Lagerstroemia* evolutionary biology and provide a foundational resource for future investigations in plant mitogenomics, phylogenetics, and horticultural biotechnology.

## Results

### *L. suprareticulata* mitochondrial complete genome assembly and annotation

**Figure 1:**
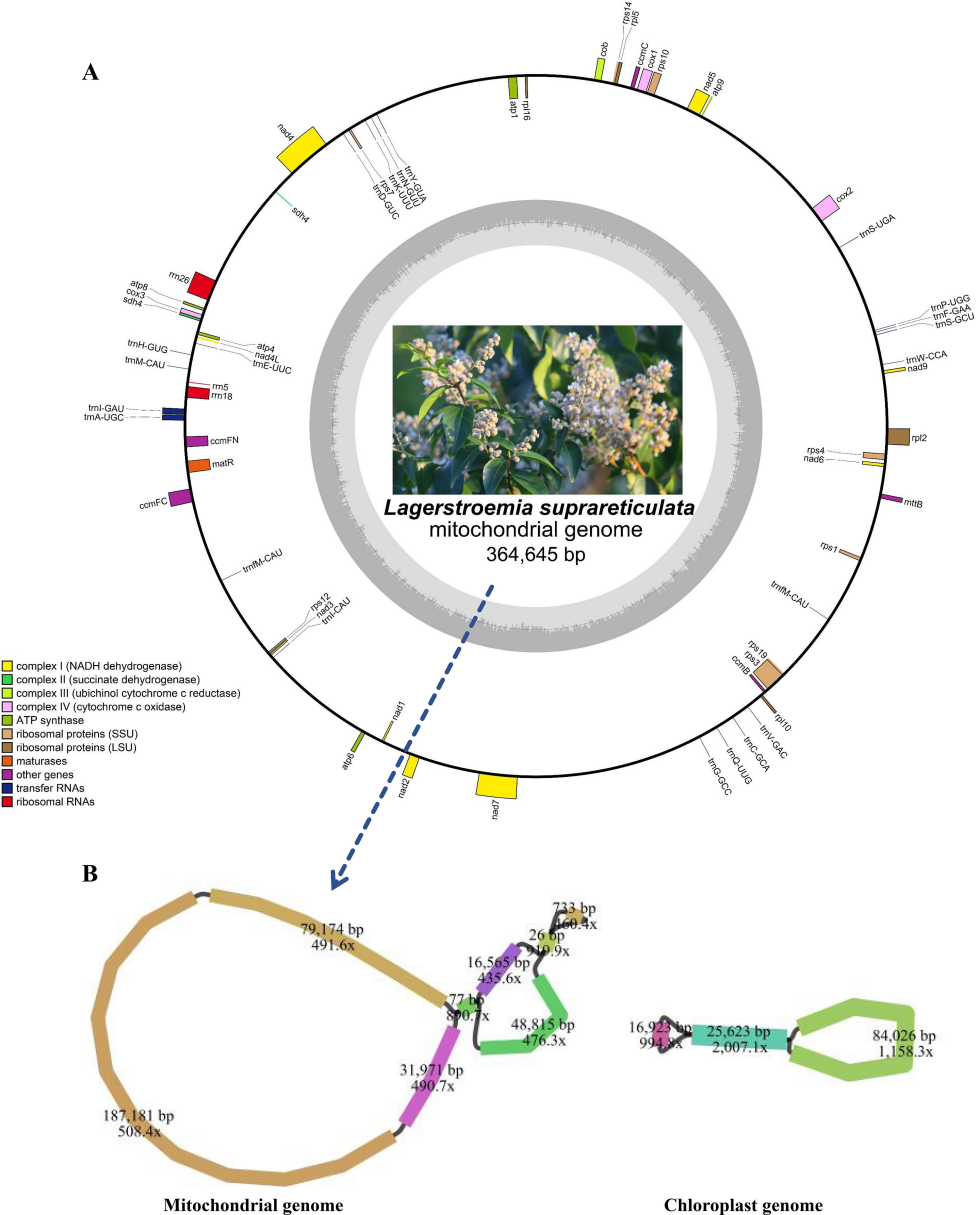
The annotated mitochondrial genomes of *L. suprareticulata*. **(A)**: Genome circle diagram **(B)**: assemblies of mitochondrial and chloroplast genomes.

**Figure 2:**
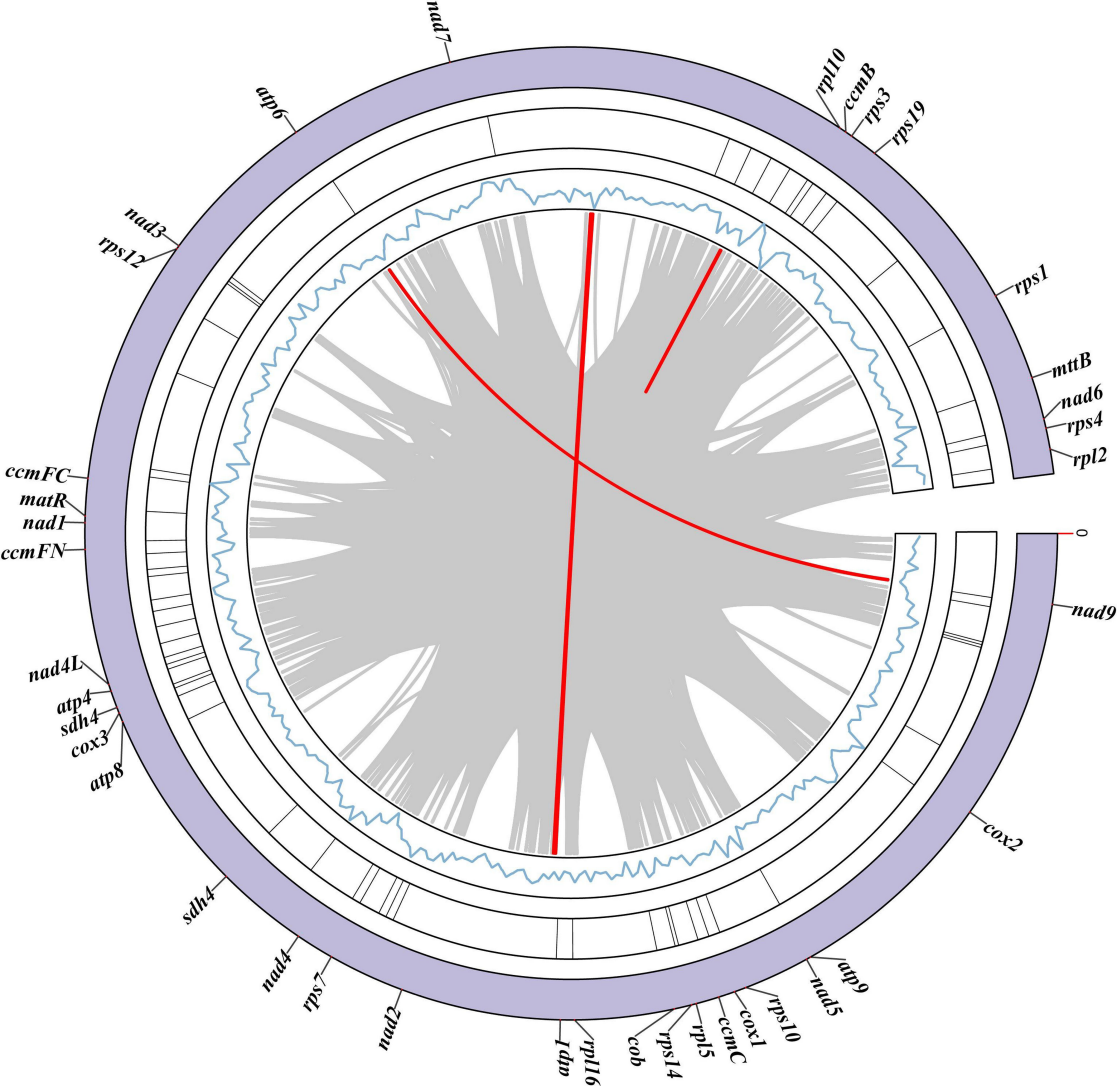
The repetitive sequence characteristics of mitochondrial genome. The inner lines of the detailed circular diagram show the repetitive sequences on each mitochondrion, with red lines marking the repetitive regions that exceed 95 bp in length.

The mitochondrial genome of *L. suprareticulata* is a single circular molecule 364,645 bp in length, with a base composition of A (26.72%), T (27.08%), C (22.93%), and G (23.28%). The GC content of the entire mitochondrial genome is 46.20% (**Figure 1**). Both genome size and GC content are similar to those of *Punica granatum* mitochondrial genome, which belongs to the same family. A total of 62 genes were located and annotated in this mitochondrial genome, with the vast majority of the sequence consisting of intergenic regions, and gene sequences accounting for only a small proportion. Among the 62 functional genes, there are 3 rRNA genes (4.84%), 21 tRNA genes (33.87%), and 38 protein-coding genes (61.29%). These 38 protein-coding genes can be further classified into 10 functional categories. With the exception of the *sdh4* gene (two copies), all other genes in the *L.suprareticulata* mitochondrial genome were present in single copy. Furthermore, 386 open reading frames (ORFs) were identified, comprising 191 on the forward strand and 195 on the reverse strand. Among these, 50 ORFs are longer than 300 bp. Repeat event analysis of the *L.suprareticulata* mitochondrial genome identified 60 repeat sequences longer than 100 bp, with the two long repeat over 500 bp, being 888 bp and 889 bp, two middle repeat, being 463 bp, respectively (**Figure 2**).

### Comparison of simple sequence repeats in *L. suprareticulata* and its related species

**Figure 3:**
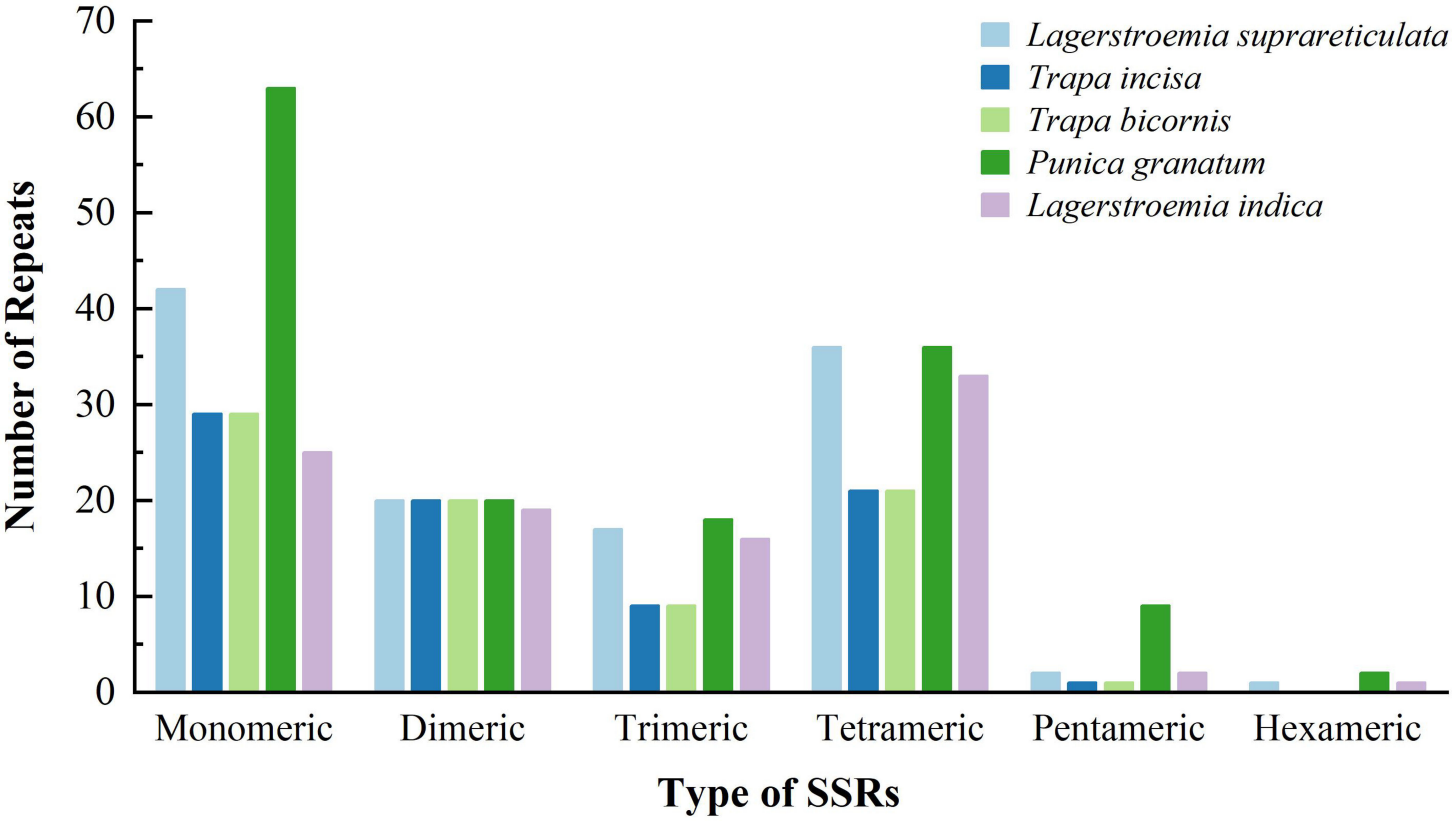
Simple sequence repeats types and numbers in the different mitochondrial genomes.

The simple sequence repeats (mtSSRs) in the mitochondrial genomes of five species within Lythraceae were identified and compared (**Figure 3**). The results showed that the mtSSRs were dominated by mono-nucleotide and tetra-nucleotide repeats. This characteristic was also observed in most of the analyzed mitochondrial genomes. Specifically, the number of mono-nucleotide repeats was 42, which was only lower than that of *P. granatum* but much higher than those of species in the genus *Trapa* and the congeneric *Lagerstroemia indica*. The number of tetra-nucleotide repeats was 36, which was comparable to that of *P. granatum* and significantly higher than those of *Trapa* species and *L. indica*. Its dinucleotide repeats were comparable to those of most species, while the abundances of penta-nucleotide and hexa-nucleotide repeats were extremely low, this result consisted with that in other species.

### Codon preference and RNA editing sites of *L. suprareticulata*

**Figure 4:**
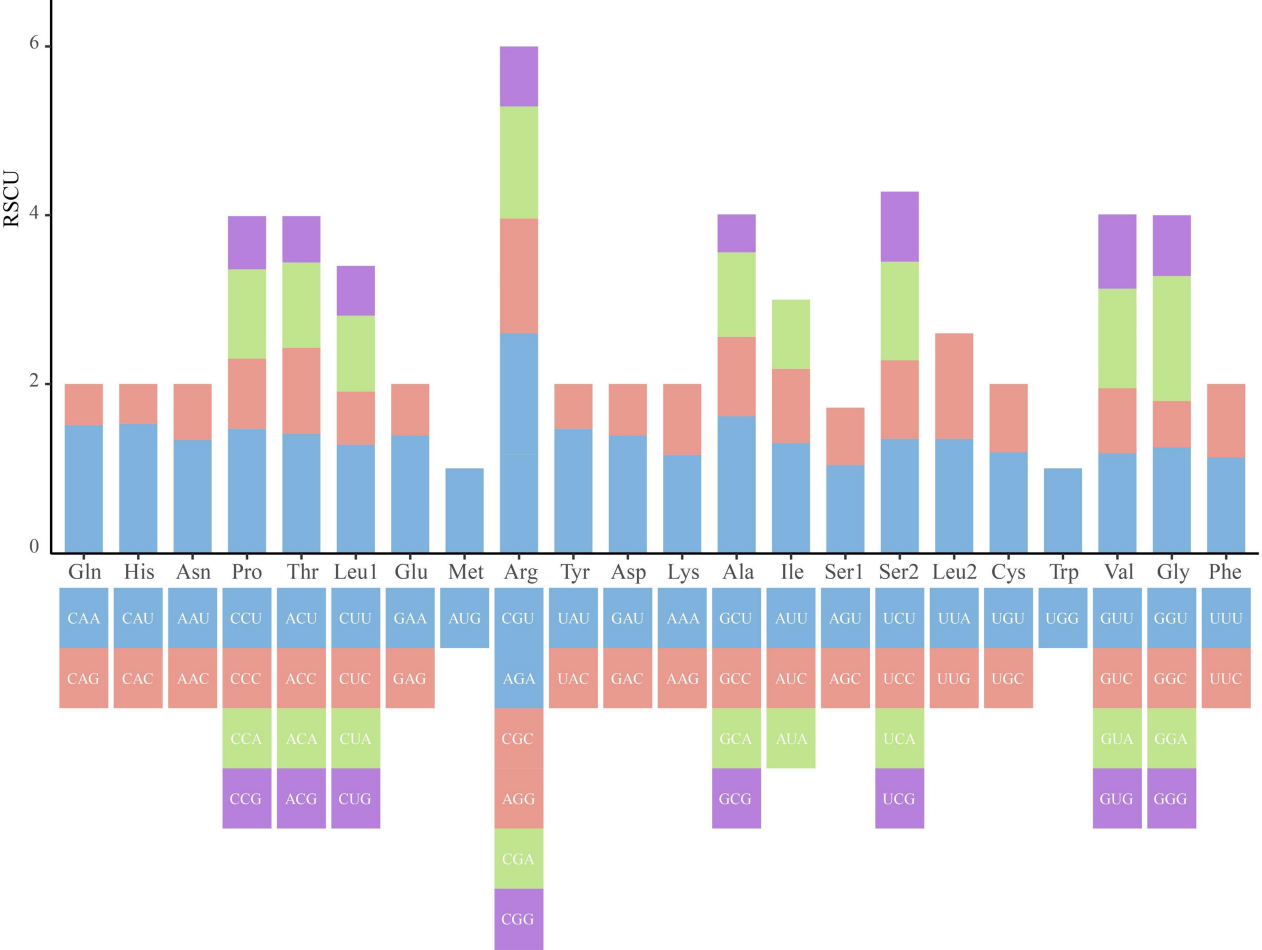
The usage of relative synonymous codons in the mitochondrial genome.

Using the default settings of Deepred-Mt software, a total of 480 potential RNA editing sites were identified in the mitochondrial genome of *L. suprareticulata*(**Figure 4**). All edits were of the C-to-U type and were distributed in 36 mitochondrial protein-coding genes. Among them, the *nad4* gene contained the largest number of RNA editing sites (41), followed by the *ccmB* gene with 30 sites. Notably, no predicted RNA editing sites were detected in the mitochondrial *rpl2* gene. Analysis of the codon usage bias of mitochondrial protein-coding genes revealed that only the relative synonymous codon usage (RSCU) values of AUG (methionine) and UGG (tryptophan) were 1, while the RSCU values of all other codons were greater than 1. Among these, GCU had the highest RSCU value (1.62), followed by CAU (1.53). In contrast, the RSCU values of GCG and CAG were both less than 0.5, indicating their relatively low codon usage frequencies.

### Analysis of genetic variations in *L. suprareticulata*

**Figure 5:**
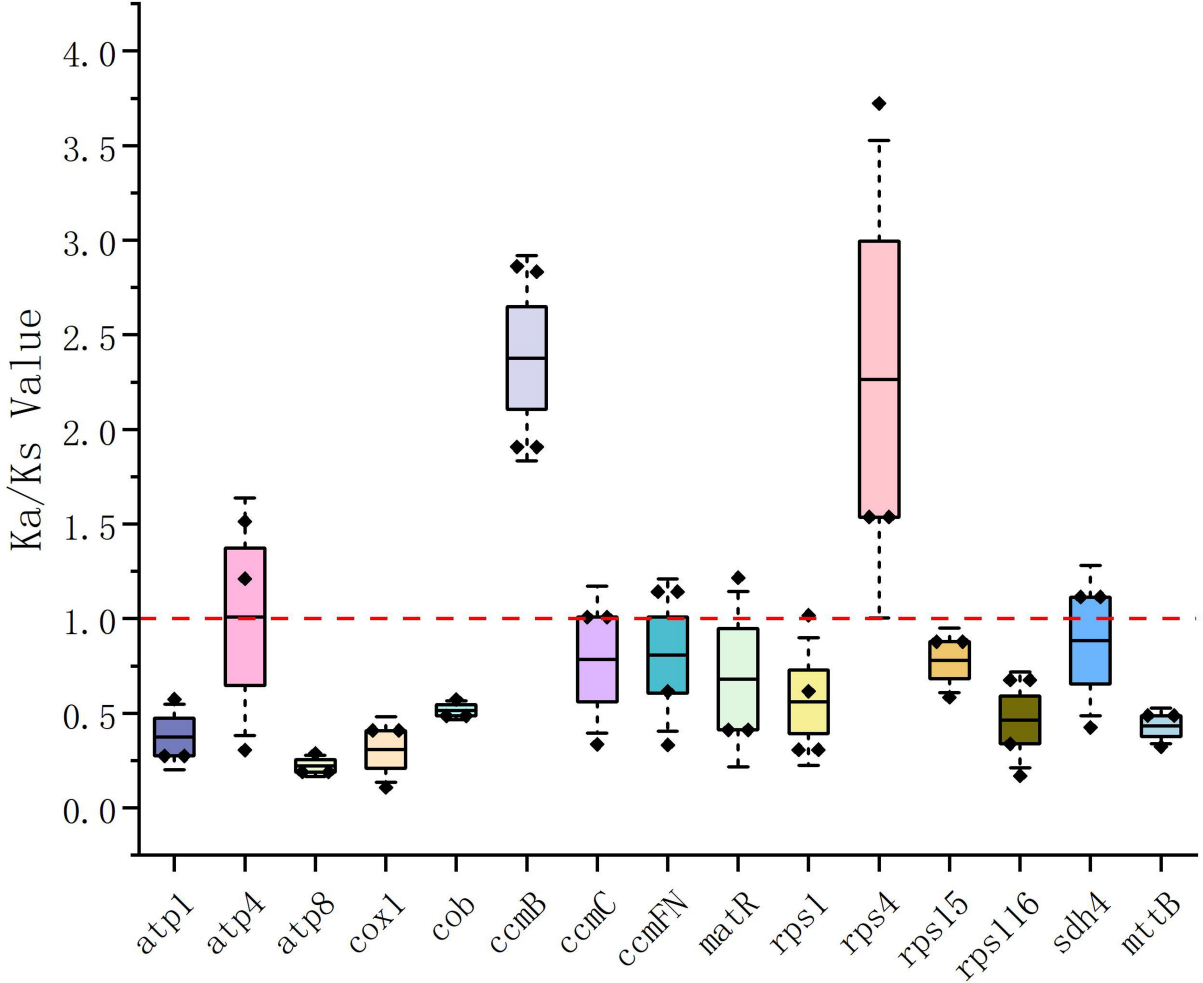
Ka/Ks values for protein-coding genes in nearby plants with *L. suprareticulata*.

The Ka/Ks values were calculated based on 15 PCGs shared by all selected plants. As shown (**Figure 5**), the Ka/Ks ratios of the *ccmB* and *rps4* genes in four species of the Lythraceae family (*Punica granatum, Trapa incisa, Trapa bicornis*, and *Lagerstroemia indica*) exceeded 1. The Ka/Ks ratios of most other genes were relatively low (approximately 0.2 to 1.0), they might have undergone a purifying selection that the *ccmB* and *rps4* genes may exhibit unique stress resistance when facing selective pressure. On the contrary, the average Ka/Ks values of *atp8* and *cox1* were the lowest among all genes (about 0.2), indicating a most obvious purifying selection for these genes. They have shown extremely high conservation during the evolution of PCGs in the plant mitochondrial genome.

### Phylogenetic evolution and sequence collinearity

**Figure 6:**
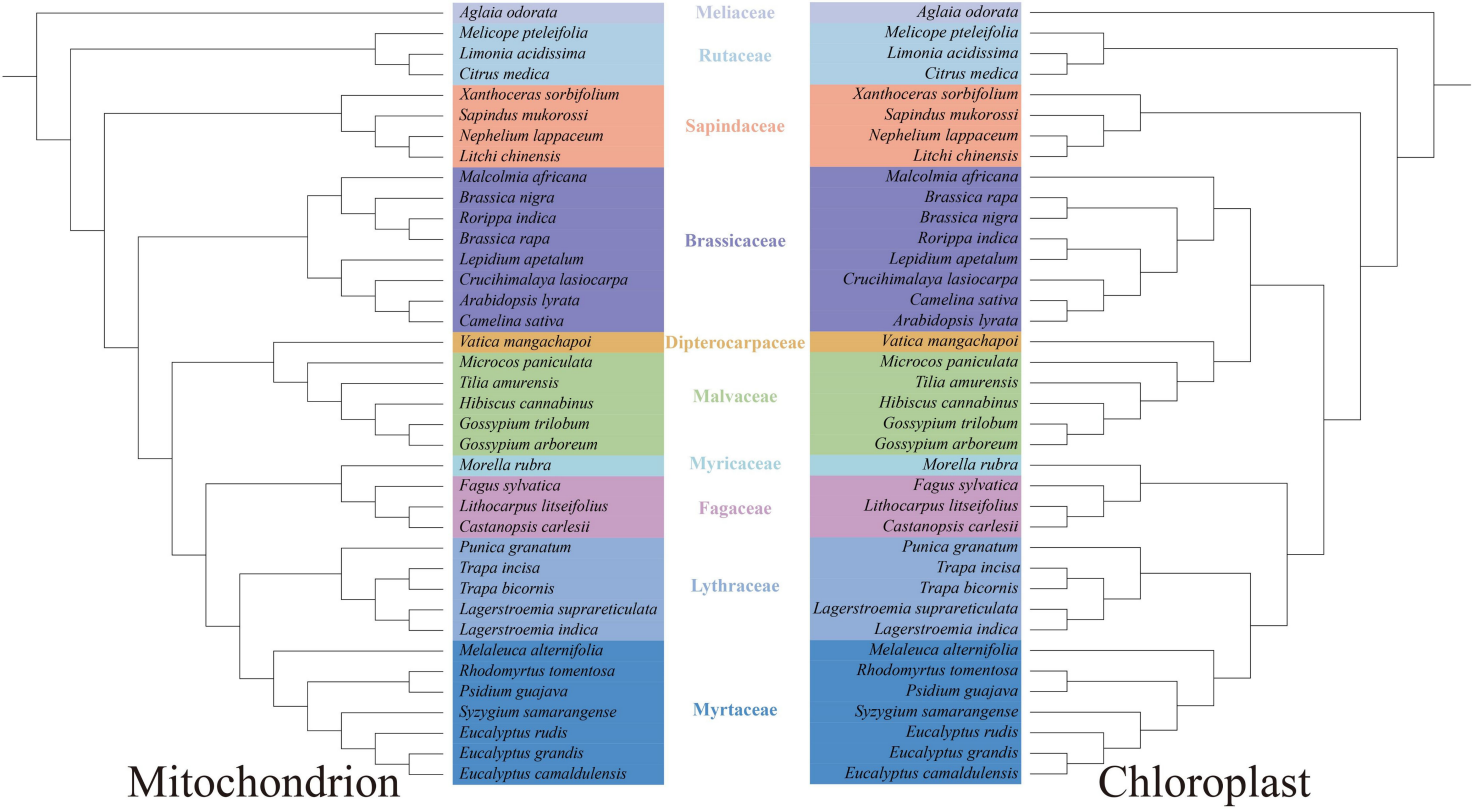
System evolution and sequence collinearity. Phylogenetic reconstruction based on conserved genes from mitochondrial and chloroplast genomes(Accession numbers: *Trapa incisa* NC_057989.1; *Lagerstroemia indica* NC_030484.1; *Trapa bicornis* NC_049010.1; *Punica granatum* NC_035240.1; *Rhodomyrtus tomentosa* NC_043848.1; *Melaleuca alternifolia* MN310606.1; *Eucalyptus camaldulensis* NC_022398.1; *Eucalyptus grandis* NC_014570.1; S*yzygium samarangense* NC_060657.1; *Psidium guajava* NC_033355.1; *Eucalyptus rudis* PP920097.1; *Tilia amurensis* NC_028588.1; *Gossypium arboretum* NC_016712.1; *Vatica mangachapoi* NC_041485.1; *Microcos paniculata* PP003957.1; *Gossypium trilobum* NC_033397.1; *Hibiscus cannabinus* NC_045873.1; *Sapindus mukorossi* NC_025554.1; *Xanthoceras sorbifolium* NC_037448.1; *Aglaia odorata* NC_048994.1; *Melicope pteleifolia* NC_050882.1; *Citrus medica* NC_050939.1; *Nephelium lappaceum* NC_053699.1; *Litchi chinensis* NC_035238.1; *Limonia acidissima* NC_086954.1; *Brassica rapa* NC_040849.1; *Lepidium apetalum* NC_051540.1; *Crucihimalaya lasiocarpa* NC_049612.1; *Malcolmia africana* NC_050827.1; *Arabidopsis lyrata* NC_034365.1; *Brassica nigra* NC_030450.1; *Camelina sativa* NC_029337.1; *Rorippa indica* NC_065833.1; *Castanopsis carlesii* NC_057119.1; *Fagus sylvatica* NC_041437.1; *Morella rubra* NC_035006.1; *Lithocarpus litseifolius* NC_063927.1).

Phylogenetic trees were constructed for the mitochondrial and chloroplast genomes of 38 species representing diverse taxonomic positions, with *Aglaia odorata* set as the outgroup (**Figure 6**). The resulting tree indicated a close evolutionary relationship between *L. suprareticulata* and *L. indica*, both belonging to the same genus. The phylogenies inferred from chloroplast and mitochondrial genomes were largely congruent and aligned with the established evolutionary relationships among angiosperm species. Nevertheless, subtle discrepancies in the internal relationships of Brassicaceae species were found between the trees inferred from mitochondrial and chloroplast data. These inconsistencies may stem from incomplete lineage sorting, where differences in evolutionary rate and sequence conservation across genomic regions lead to inferred phylogenies that do not fully reflect the actual evolutionary relationships.

**Figure 7:**
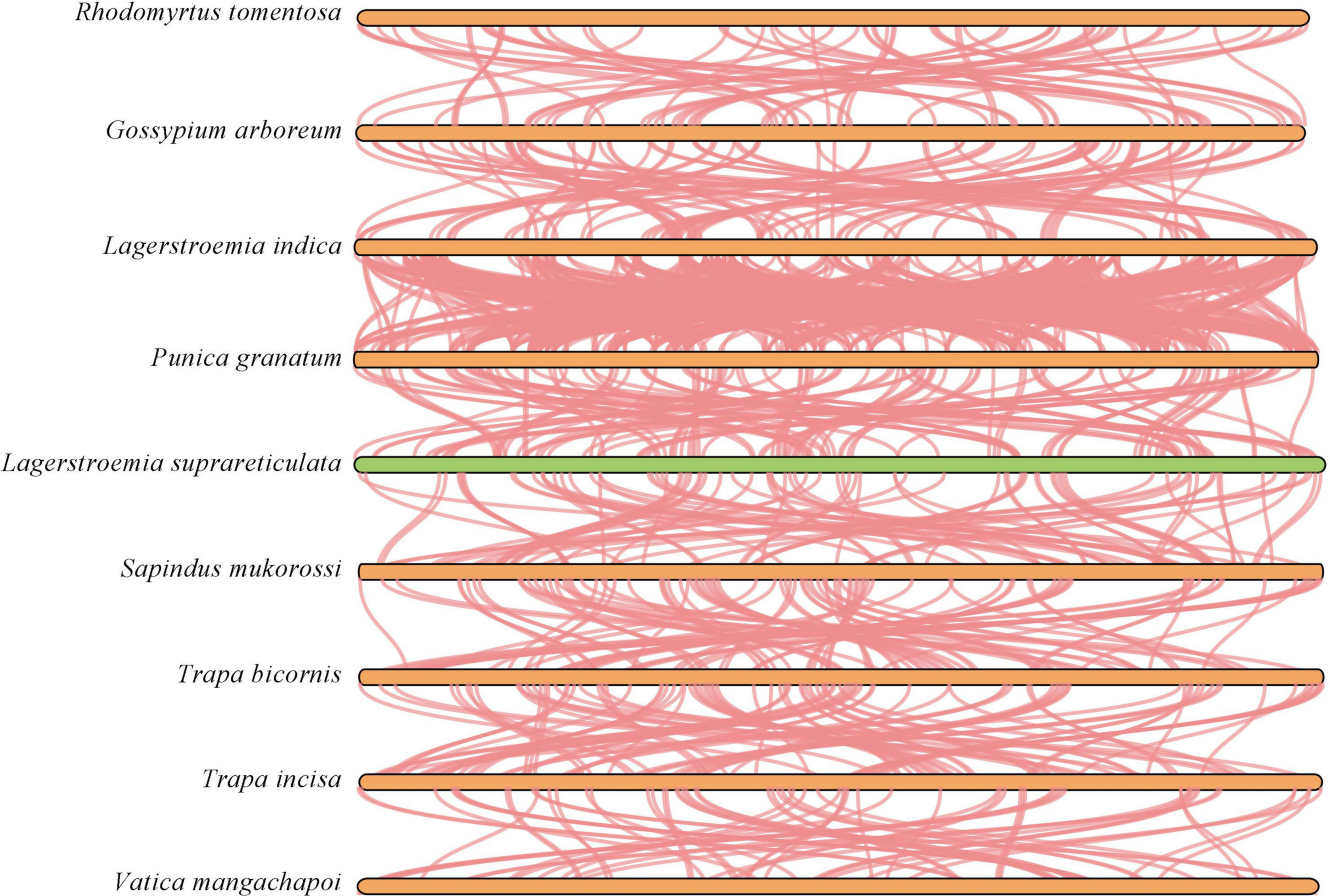
Comparative analysis of fragment collinearity in mitochondrial genomes across multiple species.

To explore collinearity across species from different phylogenetic positions, we also performed a comparative analysis of mitochondrial genomes from nine species, including *L. suprareticulata* (**Figure 7**). The dataset included *Trapa incisa, L. indica, Trapa bicornis, Punica granatum*, and *L. suprareticulata* from Lythraceae; *Rhodomyrtus tomentosa* from Myrtaceae; *Gossypium arboreum* from Malvaceae; *Vatica mangachapoi* from Dipterocarpaceae; and *Sapindus mukorossi* from Sapindaceae. The analysis revealed that *L. suprareticulata* exhibited collinear segments with all the selected species, but these blocks were mostly short. Among these species, *L. suprareticulata* displayed the strongest collinearity with *L. indica*, a species from the same genus, with the most collinear blocks identified. Fewer collinear fragments were detected between *L. suprareticulata* and the more distantly related species. The decreasing number of collinear segments with increasing evolutionary distance indicates a high level of mitochondrial genome variation across species.

### Gene rearrangement in *L. suprareticulata* and Lythraceae species

**Figure 8:**
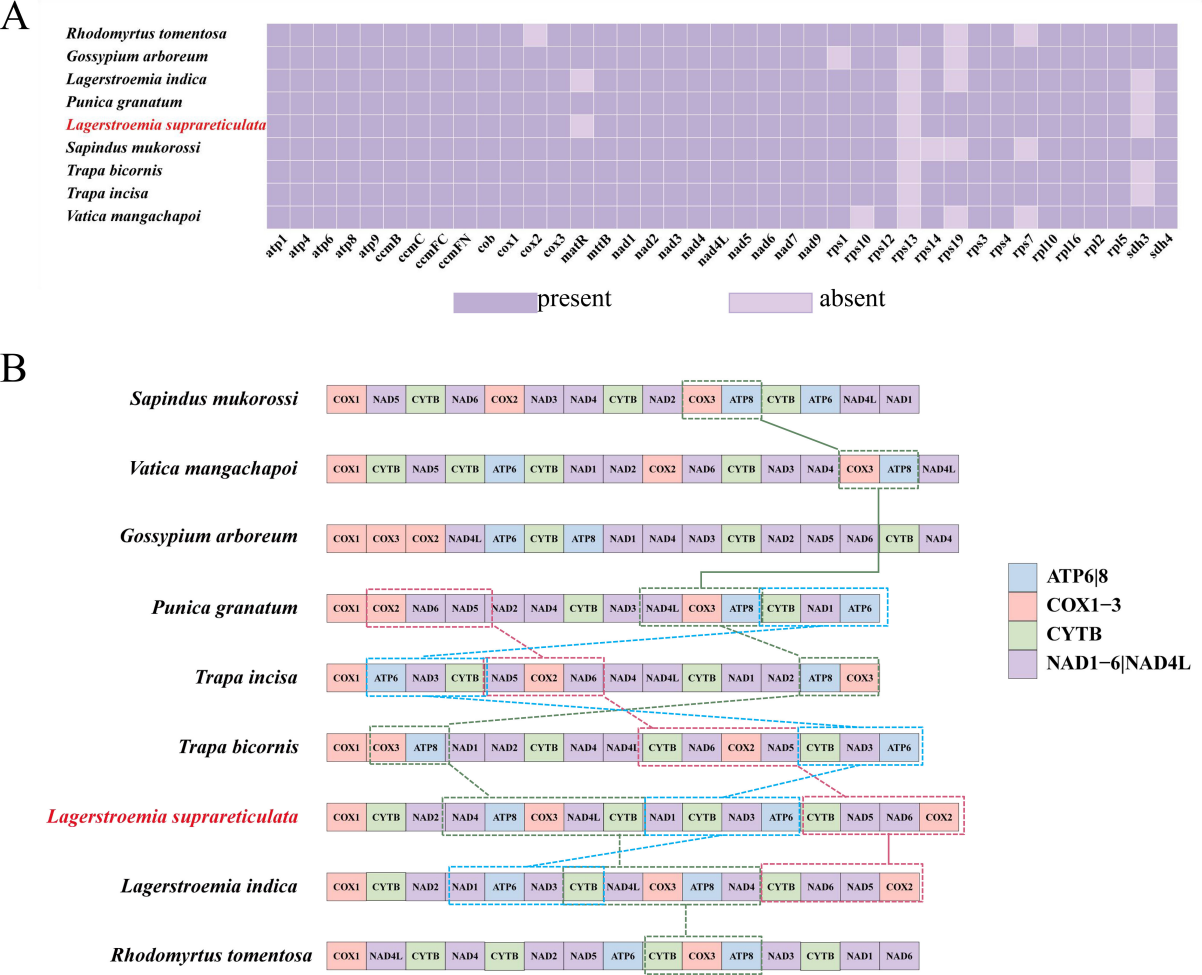
Conservation and distribution of mitochondrial genes in *L. suprareticulata* and its closely related species. **(A)** Appearance of protein-coding genes in the mitogenomes of *L. suprareticulata* and other selected species. Purple boxes indicate the presence of a gene, whereas white boxes indicate its loss in the corresponding mitogenome. **(B)** Gene rearrangements across 9 closely related species.

Mitochondrial genome structures exhibit substantial divergence across species, resulting in widespread gene rearrangement phenomena. We compared the gene arrangement pattern of the *L. suprareticulata* mitochondrial genome with four species from the Lythraceae family (*P. granatum*, *T. incisa*, *T. bicornis*, *L. indica*) and four other Magnoliopsida plants *(S. mukorossi, V. mangachapoi, G. arboreum*, and *R. tomentosa*) (**Figure 8**). The results reveal significant interspecific variation in the arrangement of the *COX3-ATP8* segment. This region is relatively conserved in *S. mukorossi, V. mangachapoi, P. granatum*, and *T. bicornis*, whereas in the genus *Lagerstroemia*, a distinct *NAD4-ATP8-COX3-NAD4L* arrangement pattern is observed, accompanied by an inversion in two species. The *CYTB-NAD3-ATP6* arrangement pattern remains relatively consistent among the closely related species *T. incisa, T. bicornis, L. suprareticulata*, and *L. indica*, although inversion or translocation events have occurred during evolution. These differences indicate that species-specific gene rearrangements have occurred in the mitochondrial genomes of Lythraceae plants during evolution. Furthermore, the detailed variations in gene arrangement patterns among different species within the family (such as between *L. suprareticulata* and *L. indica*) further reflect the dynamic and diverse nature of mitochondrial genome evolution.

### Homologous sequence analysis between the mitochondrial and chloroplast genomes of *L. suprareticulata*

**Figure 9:**
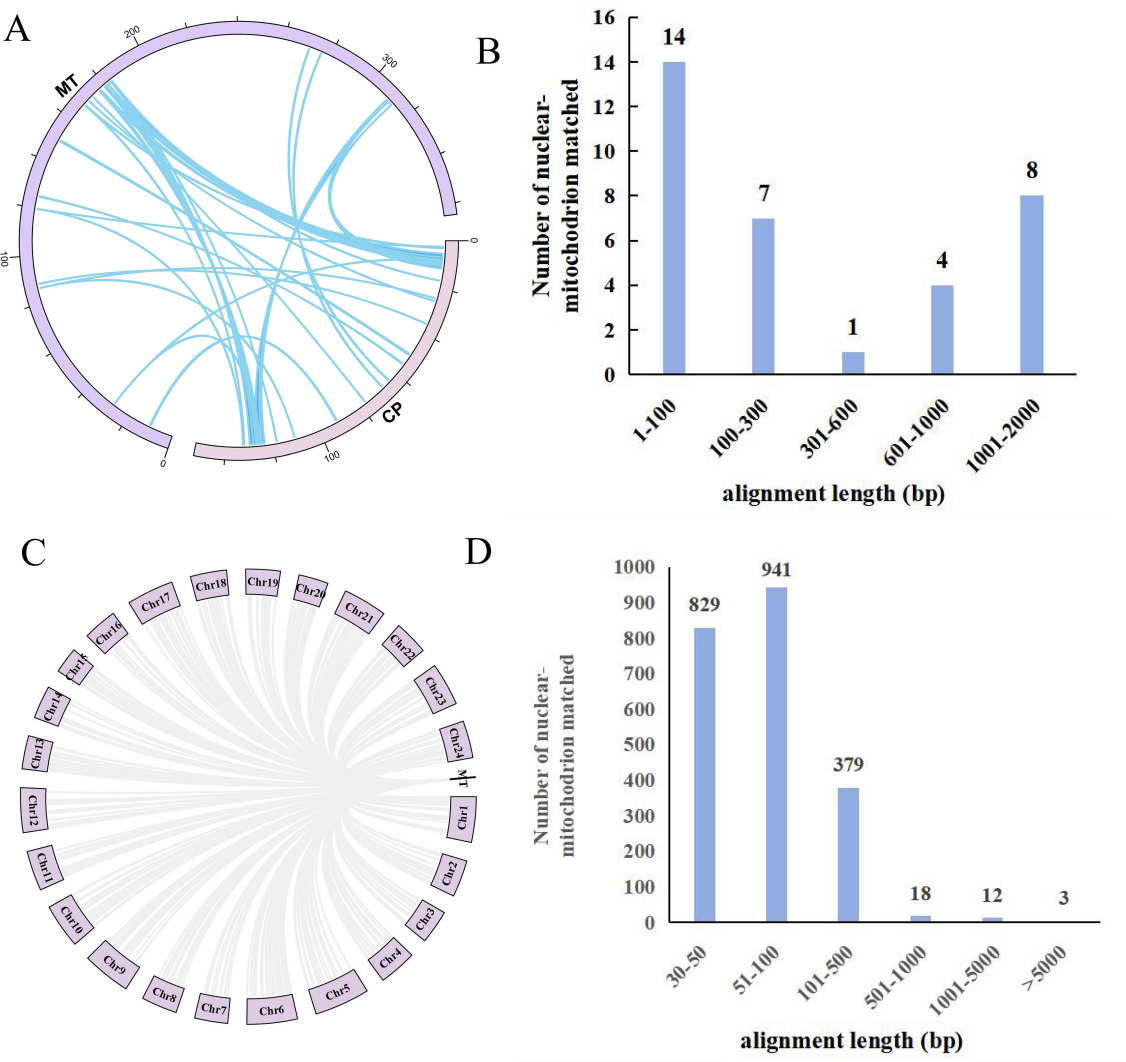
Homologous sequence between the mitochondrial, chloroplast and nuclear genomes in *L. suprareticulata*. **(A)** Homology fragments between the mitochondrial and chloroplast genomes. **(B)** Length statistics of homologous sequences between the chloroplast and mitochondrial genomes. **(C)** Homology analysis between the mitochondrial and nuclear genomes. **(D)** Fragment statistics distribution of homologous sequences between the mitochondrial and nuclear genomes of *L. suprareticulata*.

When comparing the mitochondrial and chloroplast genomes of *L. suprareticulata*, we identified 34 homologous sequences (**Figure 9A**), with a total length of 16,866 bp, accounting for 4.63% of the mitochondrial genome. Among these, homologous sequences of lower than 100 bp were the most abundant, totaling 14, followed by sequences of 1001–2000 bp, which amounted to eight (**Figure 9B**). The longest sequence was 1,485 bp, while the shortest was 33 bp. In addition, a total of seven homologous sequences were detected in the mitochondrial genome. Through comparison between the mitochondrial and nuclear genomes, we identified 2,182 homologous sequences, with a total length of 230,716 bp (**Figure 9C**). Among these, sequences of 51–100 bp were the most numerous, totaling 943, followed by sequences of 30–50 bp, which amounted to 829 (**Figure 9D**).

## Discussion

The structure and function of plant mitochondrial genomes have always been the core difficulties in current research. The structural organization of the *Lagerstroemia* mitogenome exhibits both conserved and lineage, its specific characteristics are relative to known angiosperm mitogenomes (Hu et al., 2023). Its size, which falls within the range observed for most dicotyledonous plants, is primarily driven by expansive intergenic regions and the presence of repetitive sequences, these features align with the general trend of plant mitogenome gigantism and complexity (Wu et al., 2022). Notably, the identification of multiple large inverted repeats and tandem repeat clusters within the *Lagerstroemia* mitogenome suggests that recombination-mediated rearrangements may play a pivotal role in genome plasticity, as has been documented in other plant groups (Ma et al., 2022; Sanchez-Puerta et al., 2023; Huang et al., 2025). Such structural variations could contribute to phenotypic diversity and adaptive responses to environmental stress, a hypothesis warranting further functional validation. Sloan et al. in their work on plant mitochondrial genome diversity emphasized the significance of repeat-mediated recombination in driving genomic changes (Sloan et al., 2012). These rearrangements have been associated with various aspects of plant evolution, and in the case of *Lagerstroemia*, could potentially be linked to its adaptation to different ecological niches.

Gene content analysis revealed a core set of mitochondrial genes conserved across angiosperms, including those encoding subunits of the respiratory chain complexes and ribosomal RNAs (Fučíková et al., 2014). However, the presence of several lineage-specific gene losses and pseudogenization events distinguishes the *Lagerstroemia* mitogenome from its relatives in the Lythraceae. For instance, the loss of rpl3 and truncation of sdh3 observed in our assembly mirror patterns reported in certain genera of the Myrtales, suggesting convergent evolutionary pressures on mitochondrial gene repertoires (Wang et al., 2021; Yu et al., 2024). These gene losses may reflect functional redundancy or compensation by nuclear-encoded homologs, highlighting the dynamic interplay between mitochondrial and nuclear genomes in maintaining cellular homeostasis (Adams et al., 2002). The evolution of mitochondrial gene content in plants and gene losses can occur independently in different lineages due to various evolutionary forces. In the context of *Lagerstroemia*, these losses might have been influenced by its unique evolutionary history and the functional requirements of its mitochondria (Wang et al., 2023).

Comparative mitogenomic analyses with related taxa within the Lythraceae and outgroups provide compelling evidence for distinct evolutionary trajectories. The high degree of sequence divergence in non-coding regions, coupled with conserved synteny of core genes, supports the notion that plant mitogenomes evolve through a combination of stabilizing selection on essential loci and rapid drift in intergenic regions (Cao et al., 2023; Xu et al., 2023). Phylogenetic reconstructions based on concatenated mitochondrial gene sequences robustly place *Lagerstroemia* within the Lythraceae, resolving ambiguities in previous nuclear and plastid-based phylogenies. The mitogenomic tree topology aligns with morphological classifications but reveals a closer relationship with *Punica granatum* than previously inferred (Feng et al., 2023), suggesting that mitochondrial markers may provide complementary phylogenetic signals for resolving deep divergences in this clade (Liu et al., 2012; Grosser et al., 2023). Wolfe et al. reported the concept of differential evolution in mitochondrial genomes, with essential genes being under strong selection and non-coding regions evolving more freely (Wolfe et al., 1987). This concept has been widely accepted and applied in understanding the evolutionary patterns of various plant groups, including *Lagerstroemia*.

Mitochondrial SSR (Simple Sequence Repeat) markers, leveraging their maternal inheritance and moderate polymorphism, hold unique value in plant genetic research (de Freitas et al., 2021). They facilitate the elucidation of maternal population dispersal routes (e.g., tracking southward dispersal along the Alps in Norway spruce), precise identification of crop cytoplasmic male sterility (CMS) lines, and discrimination of closely related species/varieties (e.g., differentiating *L. suprareticulata* from *L. indica* in the *Lagerstroemia* genus(**Figure 3**)). Moreover, the abundance of mtSSRs varies among plant groups (higher in monocots like rice than in dicots like *Lagerstroemia*), repeat type preferences (mononucleotide repeats dominate in angiosperms, while dinucleotide repeats are prevalent in bryophytes), and polymorphism levels (higher in woody plants like pine than in herbaceous plants like wheat). Therefore, their functional positioning and group-specific characteristics must be clearly defined in accordance with research objectives when applied.

Repetitive sequences, including tandem repeats and transposable element-like fragments constitute a significant portion of the *Lagerstroemia* mitogenome, consistent with their role as drivers of genome expansion and rearrangement (Goremykin et al., 2012). The identification of conserved repeat motifs shared with other Lythraceae species suggests ancestral origins, while lineage-specific repeats may have emerged through *de novo* evolution or horizontal transfer (Wu et al., 2015). Horizontal gene transfer (HGT) events (Stegemann et al., 2003, 2012), remain a plausible mechanism for introducing foreign DNA into the *Lagerstroemia* mitogenome, as observed in numerous plant lineages. Future investigations utilizing long-read sequencing and comparative genomics across *Lagerstroemia* species may uncover evidence of HGT and its impact on genome evolution.

## Materials and methods

### Plant materials and assembly sequence collection

*L. suprareticulata* were collected and planted in Nanning City, Guangxi, China (Lat. 22.921932° N, Lon.108.353726° E), the process of plant collection and propagation strictly complies with local government regulations, and no destructive collection has been made on the plants themselves. All the voucher specimens were kept by the local laboratory, and can be obtained with the accession number (Holotype: 2025LS0001, isotype: 2025LS0002). Fresh *L. suprareticulata* samples including leaves stems and flowers were collected from a propagate three-year-old seedlings by cuttings. (May 2022 to October 2023). All the sequenced materials, tender leaves, were surface-cleaned by 70% alcohol to remove dirt and debris. Then they were rapidly frozen in liquid nitrogen for next experiments. High-molecular-weight genomic DNA was isolated from target samples using a modified CTAB method, with residual RNA removed by RNase A digestion. The quantity and integrity of purified DNA were assessed using both of the Qubit fluorometer and agarose gel electrophoresis. Only DNA samples around 10kb were used for subsequent library preparation. SMRTbell template libraries were constructed following the manufacturer’s standard protocol for PacBio HiFi sequencing. After damage repair, end-prep and adapter ligation, the libraries were subjected to primer annealing and polymerase binding. Sequencing was conducted on a PacBio Sequel II/IIe system, and circular consensus sequencing (CCS) was applied to generate highly accurate HiFi reads. Raw sequencing data were processed using official PacBio bioinformatics pipelines to remove low-quality reads and adapters, yielding clean HiFi reads for further bioinformatic analysis.

### The direct mitochondrial genome assembly and gene annotation

The mitogenome was assembled by two approaches to guarantee accuracy. Initially, the PMAT was used for first mitogenome assembly, with the settings as ‘-st HiFi -g 300M’ (Bi et al., 2024). The second assembly was performed using Canu with default parameters (Koren et al., 2017). The two data were compared by minimap2, the final assembly was confirmed through comparison. For the convenience of description, we processed mitogenome into a linear molecule by utilizing bandage v 0.8.1 (Wick et al., 2015) to expand the common snippets. The mitogenomes were annotated using the web-based tool PMGA (www.lkmpg.cn/mgavas). Subsequently, the tRNA and rRNA genes were further examined using BLASTn, respectively. We also assembled the chloroplast genome of *L. suprareticulata* and annotated it with CPGAVAS2 (http://47.96.249.172:16019/analyzer/home) to obtain gbf files (Shi et al., 2019). The final graphs were manually checked.

### SSR site and repeat fragment detection

Simple sequence repeats (SSRs) were fast calculated by the MISA online platform (https://webblast.ipk-gatersleben.de/misa/) (Beier et al., 2017). The specified parameters were same as the previous research (Zhou et al., 2023c), encompassing mono-, di-, tri-, tetra-, penta- and hexa-nucleotides with a minimum occurrence of 10, 5, 4, 3, 3 and 3, respectively. Dispersed repeat was calculated based on a local BLAST (Altschul et al., 1990), and a cutoff of e-value 1-e^-5^ was applied. Links for repeat sequences were visualized in TBtools functions (Chen et al., 2023).

### RSCU in codons, RNA editing sites and Ka/Ks analysis

The synonymous codons (RSCU) were calculated by Phylosuite function (Zhang et al., 2020) by default parameters. PREPACT3.0 was used to predict the RNA editing sites of PCGs in the mitochondrial genomes. The threshold was established at 0.8 to minimize the similarity score in the plagiarism detector. Using TBtools Ka/Ks calculator function, we calculated the synonymous mutation rates, non-synonymous mutation rates, and their ratios (Ka/Ks).

### Phylogenetic tree construction for species with *L. suprareticulata* and comparative analysis

The other mitochondrial genomes except *L. suprareticulata* were retrieved and downloaded from NCBI for further comparison, data were obtained by April 2024 (37 species including 11 in Myrtales: *Trapa incisa* NC_086691, *Lagerstroemia indica* NC_035616, *Trapa bicornis* NC_086690, *Punica granatum* NC_071229, *Rhodomyrtus tomentosa* NC_071968, *Melaleuca alternifolia* PP533606, *Eucalyptus camaldulensis* NC_085240, *Eucalyptus grandis* NC_040010, *Syzygium samarangense* OQ701348, *Psidium guajava* PP934632, *Eucalyptus rudis* PP920092; 6 in Malvales: *Tilia amurensis* PQ072837, *Gossypium arboreum* KR736342, *Vatica mangachapoi* PP861159, *Microcos paniculata* NC_086687, *Gossypium trilobum* NC_035076, *Hibiscus cannabinus* NC_035549; 8 in Sapindales: *Sapindus mukorossi* NC_050850, *Xanthoceras sorbifolium* MK333231, *Aglaia odorata* NC_084341, *Melicope pteleifolia* PQ221923, *Citrus medica* PQ636878, *Toona fargesii* PQ287271, *Nephelium lappaceum* PP916047, *Litchi chinensis* PP932631, *Limonia acidissima* NC_086955; 8 in Brassicales: *Brassica rapa* NC_049892, *Lepidium apetalum* NC_088489, *Crucihimalaya lasiocarpa* NC_085700, *Malcolmia africana* OQ784930, *Arabidopsis lyrata* OQ852786, *Brassica nigra* NC_029182, *Camelina sativa* PQ165104, *Rorippa indica*, PP780168; 4 in Fagales: *Castanopsis carlesii* PP853255, *Fagus sylvatica* MW771358, *Morella rubra* PP533608, *Lithocarpus litseifolius* NC_065018). To ascertain the phylogenetic location and conflict of the PCGs of selected species were extracted and conserved mitochondrial and chloroplast PCGs were screened for tree building, respectively. After alignment and trimming, a maximum likelihood (ML) method was chosen in IQ-TREE to make the basic tree structure (Minh et al., 2020), based on a default nucleotide replacement model with 1000 self-developing values. The final visualization of the phylogenetic tree was achieved through iTOL software (Letunic and Bork, 2024).

## Conclusions

The complete assembly and annotation of the *L. suprareticulata* mitogenome presented herein represent a significant advancement in the understanding of mitochondrial genome evolution within the Lythraceae and broader angiosperm lineages. For the first time, our study provides a comprehensive characterization of *L. suprareticulata* mitogenome. From a practical perspective, the mitogenomic resources generated in this study offer valuable tools for horticultural research and breeding. The identification of polymorphic regions and repetitive elements provides potential molecular markers for germplasm characterization, hybrid authentication, and marker assisted selection. Additionally, insights into mitochondrial - nuclear interactions may inform strategies for enhancing stress tolerance. In conclusion, the *L. suprareticulata* mitogenome revealed novel insights into structure, evolution, and phylogenetic significance. The findings underscore the dynamic nature of plant mitochondrial genomes and their utility in addressing fundamental questions in evolutionary biology and horticulture. By establishing a foundation for future research, this work contributes to our broader understanding of angiosperm mitogenomics and enhances the practical toolkit for *Lagerstroemia* conservation and improvement.

## Data Availability

The original contributions presented in the study are included in the article/**Supplementary Material**. Further inquiries can be directed to the corresponding author.
